# Traumatic lingual ulceration in a newborn: Riga-Fede disease

**DOI:** 10.1186/1824-7288-38-20

**Published:** 2012-05-23

**Authors:** Erik H van der Meij, Tjalling W de Vries, Henk F Eggink, Jan GAM de Visscher

**Affiliations:** 1 Department of Oral and Maxillofacial Surgery, Medical Center Leeuwarden, P.O. Box 888, 8901 BR, Leeuwarden, The Netherlands; 2 Department of Paediatrics, Medical Center Leeuwarden, Leeuwarden, The Netherlands; 3 Departmant of Pathology, Medical Center Leeuwarden, Leeuwarden, The Netherlands; 4 Department of Oral and Maxillofacial Surgery, Medical Center Leeuwarden, Leeuwarden, The Netherlands

**Keywords:** incisor, infant, natal teeth, oral ulcer, tong diseases

## Abstract

Riga Fede disease is a reactive mucosal disease as a result of repetitive trauma of the tongue by the anterior primary teeth during forward and backward movement. Although the aspect of the lesion might be impressive, its nature is relatively benign. The history and clinical features are most often so typical that there is seldom a need for addititonal histopathological examination. Riga Fede disease can most often be treated with conservative measures only.

Beside the presentation of a six-month-old boy with Riga Fede disease, the literature has been reviewed as well. From this review it can be concluded that Riga Fede disease is almost exclusively restricted to the tongue, occurs soon after birth when associated with (neo)natal teeth, has a male predilection, and is in one quarter of the cases associated with neurologic disorders. In the later case, Riga Fede disease develops after the age of 6 months.

## Background

Intra-oral tumours in infancy often cause distress in both parents and doctors. The differential diagnosis includes several serious and potential lethal diseases, but also relatively benign disorders. We describe a six-month-old newborn with a benign intra-oral, ulcerating mass mimicking malignancy. The lesion was finally diagnosed as Riga Fede disease. In this treatise, the clinical characteristics, differential diagnosis, histopathological aspects, and treatment options of Riga Fede disease, based on a literature review, will be discussed. This report aims the paediatrician to recognize this entity and to prevent unnecessary invasive procedures.

## Case report

A six-month-old boy was referred to the Department of Oral and Maxillofacial Surgery by his dentist because of an ulcerative swelling on the ventral surface of the tongue, noticed by his parents since three months. The lesion seemed not to be painful as there were no feeding difficulties. The relevant medical history did not reveal any abnormalities, especially no neurologic disorders. Family history was negative for developmental disorders and congenital syndromes. The patient did not use any medication at presentation.

**Figure 1:**
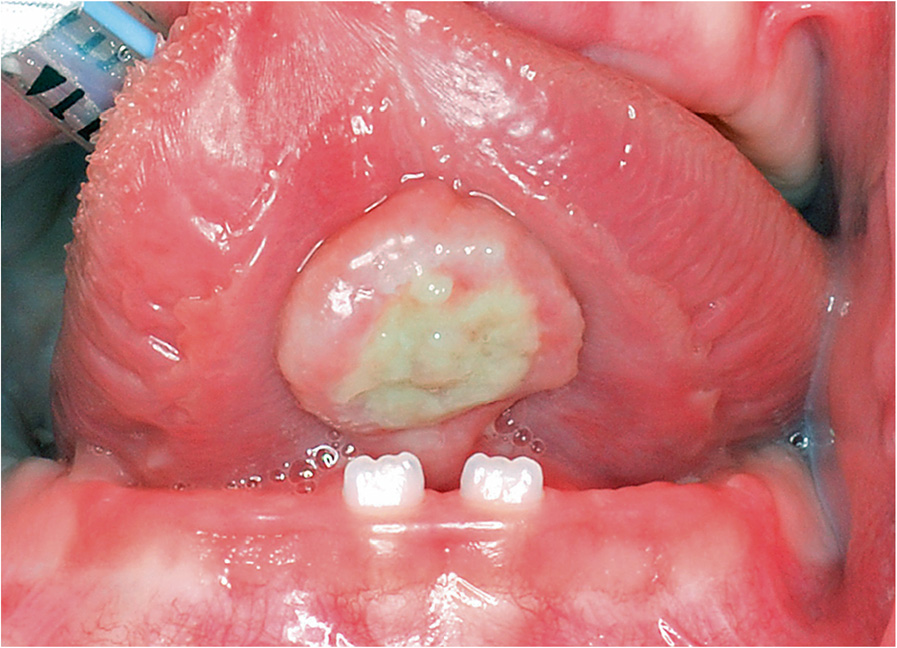
**Indurated, non-tender, ulcerative swelling on the ventral surface of the tongue measuring 1.5 by 1.5 cm.** Impressions of the primary lower central incisors were seen in the middle of the lesion.

Physical examination revealed an indurated, non-tender, ulcerative swelling on the ventral surface of the tongue measuring 1.5 by 1.5 cm. Impressions of the primary lower central incisors were seen in the middle of the lesion (Figure 1). On palpation, the lesion seemed to infiltrate deep into the underlying muscle. A close relationship between the tumor and the primary lower central incisors was noticed during swallowing.

**Figure 2:**
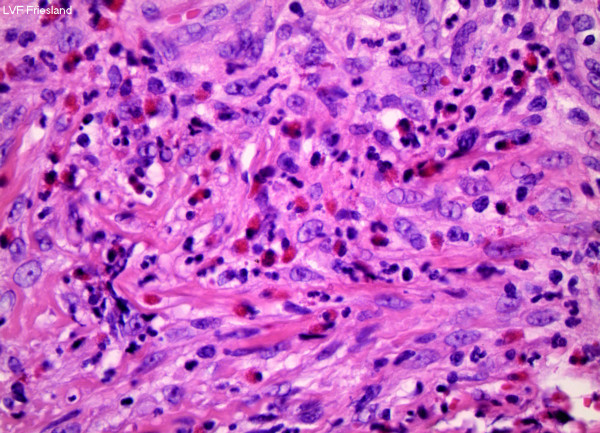
Mixed cellular infiltrate consisting of lymphocytes, neutrophils, plasma cells, and an abundant number of eosinophils (haematoxylin-eosin, 10x).

Excisional biopsy was performed under general anesthesia. The resulting defect was closed primarily. On histopathological examination an ulcerative, inflammatory lesion with granulation tissue was seen. The mixed cellular infiltrate consisted of lymphocytes, neutrophils, plasma cells, and an abundant number of eosinophils (Figure 2). Based on the clinicopathological findings a diagnosis of Riga Fede disease was made.

The tongue healed well, and three months post-operatively no signs of recurrence were found.

## Review

A literature search was performed of all cases of Riga Fede disease that were published in the English literature using the databases of PubMed, Cochrane, and Sum Search. A summary of all these reported cases is shown in Table 1[1-29].

**Table 1:** **Summary of all reported cases of patients with Riga Fede disease [**[1-29]**]**

			**AGE**	**SITE**	**(NEO)NATAL**	**BIOPSY**	**ASSOCIATED**	**TREATMENT**
**AUTHORS**	**YEAR**	**GENDER**	**(MONTHS)**		**TEETH**		**DISORDERS**	
Amberg [1]	1902	M	7	sublingual	no	yes	no	excision
Bray [2]	1927	M	9	sublingual	no	yes	no	excision
Bradley [3]	1932	F	8	sublingual	no	yes	no	excision
Moncrieff [4]	1933	M	6	sublingual	no	yes	no	weaning
Newman [5]	1935	M	6	sublingual	no	no	mentally deficient	smoothening lower incisors
		M	8	dorsum of tongue	no	no	no	extraction
Abramson [6]	1944	F	11	sublingual	no	yes	no	excision
		F	9	sublingual	no	yes	no	excision
Jacobs [7]	1956	unknown	0.3	sublingual	yes	no	no	extraction
McDaniel et al. [8]	1978	M	6	dorsum of tongue	no	yes	no	excision
Rakocz [9]	1987	M	10	base and dorsum	no	yes	FD*	composite coverage incisors
				of tongue				
Eichenfield et al. [10]	1990	F	6	sublingual	no	yes	FD*	none
Goho [11]	1996	F	0.7	sublingual	yes	no	no	extraction
		F	0.3	sublingual	yes	no	no	composite coverage incisors
Uzamiş [12]	1999	M	2	sublingual	yes	no	no	extraction
Slayton [13]	2000	M	10	sublingual	no	no	Down	
							syndrome	smoothening lower incisors
Toy [14]	2001	M	20	sublingual	no	yes	CADUPL**	unknown
				lower lip				
Baghdadi [15]	2001	M	10	sublingual	no	no	no	smoothening lower incisors
								topical corticosteroid
Baghdadi [16]	2002	F	12	sublingual	no	no	microcephaly	smoothening lower incisors
								topical corticosteroid
Terzioğlu et al. [17]	2002	M	7	sublingual	no	no	no	none
Zaenglein et al. [18]	2002	M	10	tongue	no	yes	CADUPL**	unknown
				lower lip				
Ahmet et al. [19]	2003	F	9	sublingual	no	unknown	no	none
Hegde [20]	2005	F	1	sublingual	yes	no	no	extraction
Campos-Muñoz et al. [21]	2006	M	11	sublingual	no	no	no	nasogastric feeding tube
Baroni et al. [22]	2006	M	11	sublingual	no	no	no	topical odontologic cream
								teething ring
Domingues-Cruz [23]	2007	M	24	lower lip	no	no	Down	extraction
							syndrome	
Narang et al. [24]	2008	M	9	sublingual	no	no	no	teething ring
								release of tongue tie
Jariwala et al. [25]	2008	F	1.5	sublingual	yes	no	no	extraction
Ceyhan et al. [26]	2009	M	15	sublingual	no	no	no	topical corticosteroid
Taghi et al. [27]	2009	M	8	sublingual	no	yes	cerebral palsy	composite coverage incisors
Choi et al. [28]	2009	M	8	sublingual	no	no	no	composite coverage incisors
		F	2	sublingual	yes	no	no	smoothening incisal edges
Eley et al. [29]	2010	F	11	sublingual	no	yes	no	excision
van der Meij et al.	2012	M	6	sublingual	no	yes	no	excision

* familial dysautonomia.

**congenital autonomic dysfunction with universal pain loss.

## Discussion

Riga Fede disease is a reactive traumatic mucosal disease characterized by persistent ulceration of the oral mucosa. It develops as a result of repetitive trauma of the tongue by the anterior primary teeth during forward and backward movement [26]. Although the aspect of the lesion might be impressive, its nature is relatively benign. The lesion was first described by Antonio Riga, an Italian physician, in 1881. Fede, the founder of Italian pediatrics, subsequently published histological studies and additional cases in 1890 [1]. It has therefore become known as Riga Fede disease. A broad variety of terms have been used to describe Riga Fede disease, such as eosinophilic ulcer of the oral mucosa, sublingual fibrogranuloma, sublingual growth in infants, sublingual ulcer, reparative lesion of the tongue, (neonatal) lingual traumatic ulceration, traumatic atrophic glossitis, and traumatic granuloma of the tongue.

In 1983, Elzay coined the term ‘traumatic ulcerative granuloma with stromal eosinophilia’ (TUGSE) for those chronic ulcerative lesions of the oral mucosa that histopathologically consist mainly of eosinophils [30]. As TUGSE and Riga Fede disease have the same histologic features and are often associated with a history of trauma it was suggested by Elzay that they might be considered as one entity. Although TUGSE has been mainly reported to occur in late adulthood, and not restricted in location to the tongue, it may occur in the buccal mucosa, the vestibule, gingiva, or palate, Riga Fede disease is almost exclusively restricted to the tongue. In the present literature review twenty-nine lesions appeared as ulcerations on the ventral surface of the tongue associated with repetitive trauma of the primary lower incisors, three lesions appeared on the dorsal of the tongue caused by trauma of the upper incisors, and three lesions were found on the lower lip. In seven patients the symptoms were seen soon, within two months, after birth. All these cases were associated with (neo)natal teeth. The remaining twenty-seven patients developed lesions after eruption of the lower incisors, at the age of six to twenty-four months, with a mean age of ten months. The male-to-female ratio appeared to be 1.8:1.

Riga Fede disease begins as an ulcerated area with prominent raised edges. With repeated trauma, it may progress to an enlarged, fibrous mass with the appearance of an ulcerative granuloma with superficial necrosis. Based on these characteristics the differential diagnosis of Riga Fede disease should include those entities mentioned in Table 2. Once the clinician is familiar with the diagnosis Riga Fede disease, the history and clinical features are most often so typical that there is seldom a need for addititonal histopathological examination. In the present literature review histopathological examination was performed in fourteen cases. In the remaining twenty cases a diagnosis of Riga Fede disease was made on history and clinical features alone. In our patient biopsy was performed because of unawareness of the entity of Riga Fede disease. Riga Fede disease is histopathologically characterized by an ulcerated mucosa with granulation tissue and a mixed inflammatory infiltrate consisting of lymphocytes, macrophages, mast cells and an abundant number of eosinophils, the latter being the most typical of this entity.

**Table 2:** Differential diagnosis of ulcerated, indurated masses of the oral mucosa in infancy


**LOCAL NEOPLASIA**
· granular cell tumour
· myofibroma
· sarcoma
· extra-nodal lymphoma
**INFECTION**
· congenital syphilis
· tuberculosis
**HEMATOLOGICAL DISORDER**
· agranulocytosis
**TRAUMATIC**
· mechanical (Riga Fede disease)
· electrical
· chemical

In the present literature review one quarter of the patients suffered from a neurologic disorders, i.c. familial dysautonomia, congenital autonomic dysfunction with universal pain loss, Down syndrome, microcephaly, and cerebral palsy. Interestingly, all seven patients with (neo)natal teeth developed Riga Fede disease before the age of six months and did not suffer from neurologic disorders. According to these findings Domingues–Cruz et al. proposed using a classification of the disease wherin ‘precocious Riga fede disease’ defines those occurrences associated with (neo)natal teeth in the first 6 months of life, where no relation with neurologic disorders was found, and ‘late Riga Fede disease’ refers to those instances which typically start after 6–8 months of life, with the first dentition, usually the lower incisors. In the former, the existence of (neo)natal teeth, together with the instinctive sucking reflex and the tendency for the tongue to protrude favor the development of the disease. In the latter, the importance of recognition of the condition is due to its possible relationship to neurologic disease [23].

Several treatments for Riga Fede disease have been described, all of which aim to eliminate the source of trauma so healing can take place. It is preferably to start treatment conservatively such as smoothening off the incisor edges, covering the rough incisor edges with composite resin, changing feeding habits by using a bottle with a larger hole in the nipple, placing a nasogastric tube, or relieving symptoms by application of a local corticosteroid. If conservative methods fail to resolve the lesion, or when the child is severely dehydrated or malnourished extraction of the incisors might be considered. Alternatively, excision of the lesion itself might be performed.

## Conclusion

In conclusion, Riga Fede disease is a reactive mucosal disease as a result of repetitive trauma of the tongue by the anterior primary teeth during forward and backward movement. Although the aspect of the lesion might be impressive, its nature is relatively benign. The history and clinical features are most often so typical that there is seldom a need for addititonal histopathological examination. Riga Fede disease can most often be treated with conservative measures only.

## Consent

Written informed consent was obtained from the parents/ guardians of the patient for publication of this Case report and any accompanying images. A copy of the written consent is available for review by the Editor-in-Chief of this journal.

## Competing interests

The author(s) declare that they have no competing interests.

## Authors’ contribution

All authors have equally participated in drafting of the manuscript and/or critical revision of the manuscript for important intellectual content. All authors read and approved the final manuscript.

## Funding

This research received no specific funding.

## References

[B1] Amberg S Sublingual growth in infants Am J Med Sci 1902 126 257 69

[B2] Bray CM Riga’s disease (Cachectic Aphthae) W Va Med J 1927 23 249 50

[B3] Bradley DJ Sublingual growth-Riga’s or Fede’s disease J Med 1932 13 473 74

[B4] Moncrieff A Sublingual ulcer: with special reference to Tiga’s disease Br J Child Dis 1933 30 268 74

[B5] Newman PH A case of double Riga’s disease Br J Child Dis 1935 32 39 41

[B6] Abramson M Dowrie JO Sublingual granuloma in infancy (Riga-Fede’s disease) J Pediatr 1944 24 195 98 10.1016/S0022-3476(44)80125-1

[B7] Jacobs M Oral lesions in childhood. Oral Surg 1956 9 871 81 10.1016/0030-4220(56)90354-1 13349120

[B8] McDaniel RK Morano PD Reparative lesion of the tongue Oral Surg. Oral Surg 1978 45 266 71 10.1016/0030-4220(78)90094-4 272608

[B9] Rakocz M Frand M Brand N Familial dysautonomia with Riga-Fede's disease: report of case ASDC J Dent Child 1987 54 57 9 3468143

[B10] Eichenfield LF Honig PJ Nelson L Traumatic granuloma of the tongue (Riga-Fede disease): association with familial dysautonomia J Pediatr 1990 116 742 4 10.1016/S0022-3476(05)82663-0 1691779

[B11] Goho C Neonatal sublingual traumatic ulceration (Riga-Fede disease): reports of cases ASDC J Dent Child 1996 63 362 4 8958351

[B12] Uzamiş M Turgut M Olmez S Neonatal sublingual traumatic ulceration (Riga-Fede disease): a case report Turk J Pediatr 1999 41 113 6 10770685

[B13] Slayton RL Treatment alternatives for sublingual traumatic ulceration (Riga-Fede disease) Pediatr Dent 2000 22 413 4 11048312

[B14] Toy BR Congenital autonomic dysfunction with universal pain loss (Riga-Fede disease) Dermatol Online J 2001 7 17 12165233

[B15] Baghdadi ZD Riga-Fede disease: report of a case and review J Clin Pediatr Dent 2001 25 209 13 12049080 10.17796/jcpd.25.3.9725k31q263800km

[B16] Baghdadi ZD Riga-Fede disease: association with microcephaly Int J Paediatr Dent 2002 12 442 5 10.1046/j.1365-263X.2002.00396.x 12452988

[B17] Terzioğlu A Bingül F Aslan G Lingual traumatic ulceration (Riga-Fede disease) J Oral Maxillofac Surg 2002 60 478 10.1053/joms.2002.32070 11928116

[B18] Zaenglein AL Chang MW Meehan SA Axelrod FB Orlow SJ Extensive Riga-Fede disease of the lip and tongue J Am Acad Dermatol 2002 47 445 7 10.1067/mjd.2002.117213 12196759

[B19] Ahmet T Ferruh B Gürcan A Lingual traumatic ulceration (Riga-Fede disease) Br J Oral Maxillofac Surg 2003 41 201 12804551 10.1016/s0266-4356(03)00044-5

[B20] Hegde RJ Sublingual traumatic ulceration due to neonatal teeth (Riga-Fede disease) J Indian Soc Pedo Prev Dent 2005 23 51 52 10.4103/0970-4388.16031 15858311

[B21] Campos-Muñoz L Quesada-Cortés A Corral-De la Calle M Arranz-Sánchez D Gonzalez-Beato MJ De Lucas R Vidaurrázaga C Tongue ulcer in a child: Riga-Fede disease J Eur Acad Dermatol Venereol 2006 20 1357 9 10.1111/j.1468-3083.2006.01715.x 17062075

[B22] Baroni A Capristo C Rossiello L Faccenda F Satriano RA Lingual traumatic ulceration (Riga-Fede disease) Int J Dermatol 2006 45 1096 7 10.1111/j.1365-4632.2004.02554.x 16961520

[B23] Domingues-Cruz J Herrera A Fernandez-Crehuet P Garcia-Bravo B Camacho F Riga-Fede disease associated with postanoxic encephalopathy and trisomy 21: a proposed classification Pediatr Dermatol 2007 24 663 5 10.1111/j.1525-1470.2007.00564.x 18035997

[B24] Narang T De D Kanwar AJ Riga-Fede disease: trauma due to teeth or tongue tie? J Eur Acad Dermatol Venereol 2008 22 395 6 10.1111/j.1468-3083.2007.02347.x 18269624

[B25] Jariwala D Graham RM Lewis T Riga-Fede disease Br Dent J 2008 204 171 10.1038/bdj.2008.113 18297006

[B26] Ceyhan AM Yildirim M Basak PY Akkaya VB Ayata A Traumatic lingual ulcer in a child Clin Exp Dermatol 2009 34 186 8 10.1111/j.1365-2230.2008.02796.x 19187299

[B27] Taghi A Motamedi MHK Riga-Fede disease: a histological study and case report Indian J Dent Research 2009 20 227 9 10.4103/0970-9290.52893 19553727

[B28] Choi SC Park JH Choi YC Kim GT Sublingual traumatic ulceration (a Riga-Fede disease): report of two cases Dental Traumatol 2009 25 48 50 10.1111/j.1600-9657.2009.00773.x 19583570

[B29] Eley KA Watt-Smith PA Watt-Smith SR Deformity of the tongue in an infant: Riga- Fede disease Paediatr Child Health 2010 15 581 82 22043140 10.1093/pch/15.9.581 PMC3009564

[B30] Elzay RP Traumatic ulcerative granuloma with stromal eosinophilia (Riga-Fede’s disease and traumatic eosinophilic granuloma) Oral Surg Oral Med Oral Pathol 1983 55 497 506 10.1016/0030-4220(83)90236-0 6575340

